# The Associations between Childhood Experiences and Occupational Choice Capability, and the Mediation of Societal Gender Roles

**DOI:** 10.3390/healthcare10061004

**Published:** 2022-05-29

**Authors:** Orhan Koçak, Meryem Ergin, Mustafa Z. Younis

**Affiliations:** 1 Faculty of Health Science, Istanbul University—Cerrahpasa, Istanbul 34320, Turkey; meryemergin53@gmail.com; 2 College of Health Sciences, Jackson State University, 350 W. Woodrow Wilson Dr., Jackson, MS 39213, USA; mustafa.younis@jsums.edu

**Keywords:** childhood experiences, gender roles, career choice

## Abstract

Experiences in family, school, and social life during childhood are associated with gender roles and occupational choice capability. This study examines how competent individuals are in occupational choice capability and the relationships of childhood experiences and gender roles with their competencies in occupational choice capability. The research is composed of 805 individuals aged 18 and older, who reside in Turkey. In the research, we used the Personal Information Form, Childhood Experiences Scale, Gender Roles Attitude Scale, and The Scale of Occupational Choice Capability. The SPSS 25 program and PROCESS-Macro were used to analyze the variables. The relationship between the scales was investigated using Pearson correlation analysis and multiple regression analysis. According to the findings we obtained, individuals’ family and school life were positively correlated with their career choices, and family function sexism harmed their choice of profession. We also found gender roles had a mediating role in the relationship between school life and career choice.

## 1. Introduction

Childhood experiences in family, school, and social life are linked to gender roles and professional choice ability [1,2]. Childhood is an integral part of people’s lives. People’s childhood experiences affect both adolescence and adulthood phases [3,4,5]. During this period, individuals’ positive support from their families, friends, and school affects them by being more positive and thriving throughout their lives [6,7,8]. In contrast, those who deal with negative emotions have to deal with negative situations from their childhood and are likely to experience problems in their adult life [9,10,11,12,13].

In many societies, gender roles are determined after the child’s gender is determined before birth. Parents in the family make preparations at home according to the gender of the child to be born, and even after birth, the roles of children are imposed by their families and close environments [14,15]. Therefore, children may reflect what they see in their families’ behaviors and become reflections of their parents somehow. Individuals, who take the ethical values of their family, live with these values throughout their lives [16,17,18,19,20,21,22]. Children, who see equality in the distribution of roles of the mother and father, and support each other by solidarity, will adopt them in their adult life [23,24,25].

The experiences of individuals in the family, school, and social life during their childhood may affect their gender roles and career choices in the future. Individuals primarily begin to decide what they want to be when they grow up, as children, which takes shape during adolescence [1,26]. Individuals need to choose the professions they want, suitable for their abilities, and both for their career and personal development. The choice of profession is a process that affects people’s entire lives. However, not every individual can choose the profession they wish because childhood experiences and gender roles can affect this process [27,28,29,30].

Gender roles are much more prominent in Turkish society than in the West [31,32]. But, the impact of family and gender roles changes over time as women’s education levels increase and they enter the labor markets [33,34]. Therefore, this study will contribute to the literature to understand the changing associations between childhood experiences and gender roles. A research question was asked at the beginning of the study: How are childhood experiences and gender roles related to occupational choice capability?

Individuals who do not receive enough support during childhood, and confront the pressure on men and women’s roles by society, either direct their profession choice accordingly or turn to a profession against their will. We investigated whether gender roles mediate the associations between individuals’ school life and their choice of profession. Based on the hypotheses and the current literature results, evaluations were made in the discussion and conclusion.

### 1.1. Childhood Experiences

Childhood is a period about which many people, from various fields, have worked and put forward their views. Relationships between child and parent are essential for parents, and they try to be close with their children and establish a friendly relationship [35,36,37,38]. Parents want their children to share everything with them comfortably, express their wishes and desires without worry, and have a pleasant time together. However, there are important factors that parents who try to be friends with their children should pay attention to. The most important of these elements is that the parenting role is not the friendship role. Parents should know that their children can make many friends throughout their lives, but their children will see them as parents throughout their lives and act according to that [39,40,41,42].

In identity formation, the children internalize their parents’ values until they reach adolescence [41,43]. Parents’ child-rearing attitudes have an important place in determining what personality they will have. Many researchers believe that the most influential people in individuals’ lives are the mother and father. Having a positive relationship with their parents has an essential place in adolescents’ and adults’ mental health [44].

Parents’ attitudes in a democratic family should be compatible with each other, determined, and reassuring. In this sense, allowing children to perform some behaviors within certain limits will create an environment for children’s understanding of responsibility. It is observed that children who grow up in a democratic family environment have a lower tendency to violence [45,46,47]. In an overprotective family, parents overprotect their children and supervise their every behavior. Parents intervene too much in their children’s behavior to the point that this could hinder children’s development. In authoritarian families, parents allow their children’s behavior at the level they deem suitable. Usually, perfectionist parents are seen with this attitude. However, children who grow up in authoritarian families will be unstable, can not produce new ideas, and are more prone to fear and anxiety [45,48].

Children develop relationships with other people, such as teachers and friends, as well as parents, and these play an essential role in their psycho-social development [49,50,51,52]. There is a connection between the basic sense of trust children gain in the early years and their school success. Having an accepting or affirming relationship with the child helps the child develop a responsible and self-controlling personality. On the contrary, a rejecting or disapproving relationship causes the child to display aggressive behaviors and develop insecure and shy personality traits [53,54,55].

The creativity and productivity levels of individuals who receive love, care, affection, trust, and appreciation from their family and friends during childhood also increase. Individuals who can develop healthy relationships with their family, friends, and relatives in childhood also develop a sense of autonomy, which may affect their success in business life in adulthood [56,57].

Children with a single-parent, who grow up with grandparents, or are separated from their parents are not socialized enough. Their education levels are also often low due to financial inadequacy and lack of social environment and interest [58]. Therefore, it is insufficient to only meet the attention and compassion needs of individuals during childhood. Since individuals need to feel strong in their environment, they will also need social support from relatives, friends, and parents during childhood.

The school covers a large part of the childhood life of individuals. Therefore, school life in childhood plays an essential role in the future of individuals. It is possible that individuals who are not satisfied with their school life may not be satisfied with their lives in the future [59]. Childhood school friendships can contribute to individuals’ socialization and communication skills, character development, education, and academic development [60]. However, teachers, who are the most important individuals in school life, play an important role in children’s emotional, cognitive, and academic development [61].

In order to gain control in their social lives, individuals start to develop different strategies such as avoiding, ignoring, resisting, or being social during their childhood [62]. Children who are not accepted or excluded by their peers have fewer friends than children who are accepted by their peers, and their loneliness level is higher than the others [63]. Life satisfaction of individuals increases with the social support received from their friends during childhood [64]. Therefore, childhood friends and relationships can play a decisive role in individuals’ current and future social lives and career decisions.

### 1.2. Occupational Choice (OCCS)

The profession or career choice is a vital element for individuals because the modern world revolves around it. It is implemented after intensive training that is carried out within specific rules. Individuals contribute to the life of society by carrying out their professions and then earning money in return in the modern world [65,66,67]. A profession determines the place of the individual in society, and so the choices people make in their career development process are the most critical decisions in their lives [68,69,70].

On the one hand, new technologies offer new opportunities not only for industries but also for people. On the other hand, with new technologies, how we do business and do work changes over time [71]. Rapid changes have been creating both challenges and opportunities for individuals. So in this changing world, people usually remain indecisive when choosing or changing a profession if they do not have enough support. Parents should support their children in their career decisions to eliminate these difficulties by considering children’s interests, abilities, and expectations [72,73,74,75].

Job advantages, interest in the profession, abilities, and socio-demographic characteristics come into effect in the choice of profession. Family, friends, and teachers can also be influential in an individual’s choice of profession. However, there are situations where the individual makes a career choice unconsciously without any support and plan [72,73,75]. According to Thomson et al. (2012), the individual should take career steps by analyzing the sector, having information about the advantages and disadvantages, learning the professional experiences of working, and determining whether the profession is suitable for them [76,77].

Individuals commonly choose a profession with the orientation and influence of the family and social environment, without any support and being aware of their interests, desires, abilities, and knowing which profession they are predisposed to [78]. The profession is a set of social roles and determines individuals’ lifestyles and behaviors [79,80,81,82]. In this sense, labor markets are being shaped by individuals’ career choices. If career choices are not made properly, labor markets will not work properly, and then social issues will emerge, such as unemployment, family separations, and psychological problems. Individuals need to know how to fulfill their responsibilities more efficiently. The concept of capability used in this research means special knowledge that provides the power to do something [83]. Occupational choice capability (OCCS) includes individuals choosing the right profession that best suits them, their abilities, and interests, and they have the knowledge and skills about them and their career planning [84]. Despite all this, it is also known that individuals unconsciously choose their profession without any career planning [85,86,87]. Researchers have focused on having professional knowledge and skills, employment opportunities, job security, salary, social reputation, and environmental factors such as family and friends, which impact people’s choice of a profession [73,75,88]. There will be more macro problems in communities if some issues resulting from family, school, and society’s roles are not solved.

### 1.3. Gender Roles

Gender roles are imposed on individuals from the moment they are created. The colors of their rooms, their clothes, the types and shapes of their toys, and the games that are suitable for them to play are all determined by their parents. Children are brought up on the necessity of behaving according to these norms by internalizing and making these a part of their social life and roles [14,15,89]. According to these roles, women are expected not to go out in the evening, not to work, to get married, to do housework, and take care of children, whereas men are expected to earn money for the family and to repair and renovate the house. It can be seen in societies that the man can inflict violence on his wife when necessary and that the woman remains silent against this violence [90]. While masculinity is focused on achievement and control, femininity is focused on closeness and support [91]. While girls are expected to help their mothers with housework, boys are expected to help their fathers. According to these approaches, women must adjust their social and working lives according to their spouses and children [92].

The concept of gender rejects universal definitions of men and women and states that gender is based not on biological data but is culturally constructed [14,93,94]. The feminist approach is defined as biological sexuality that separates men and women from each other. Social gender is the values, roles, behaviors, and expectations imposed on individuals by their cultures [89,93,94,95,96].

Discrimination may occur in the uptake of opportunities, resource allocation, and access to services due to the gender of the individual. Discrimination may arise in various areas, such as in receiving an education and benefiting from health and legal processes and professions [97]. Gender-based discrimination in working life differentiates women and men, affects their access to social resources, and causes inequality [98]. The inequality becomes a serious obstacle to women and society’s economic, political, and cultural development [99,100,101,102]. The roles assigned to women within the traditional values of family and society play an essential role in choosing professions. Some approaches towards girls, such as they should be passive, nurturing, sensitive, emotional, tend towards domestic activities, and cannot be successful in mathematics and engineering, are the roles defined by family and society [97,103,104].

It is observed in many countries, that women are expected to stay at home and take care of their families instead of working. As a result, they are exposed to gender-based discrimination in their social and working life [95,105]. However, many countries have been developing projects, policies, and action plans to change this situation and contribute to women’s development and democratization, in all sectors [100,102,103].

#### 1.3.1. Family Function Sexism (FFS)

Men see themselves as superior to women, so there is a view that men have the authority to restrict women’s values, roles, and lifestyles [106,107,108]. Due to the belief that men have very different and superior abilities to women in business life, it is suggested that their primary duty is to provide for themselves while their spouses take care of the housework [95]. The belief that men should do repair and maintenance, and women should carry out jobs such as childcare and cooking can also be supported by women [102,109]. As family function sexism increases, individuals may ignore or excuse domestic violence [110].

#### 1.3.2. Family Function Transcendent (FFT)

It can be observed that as liberalism develops, so does the transcendent function of the family [111]. Fathers play a more active role in housework in egalitarian families [112]. In addition, there is a widespread belief in developed countries that women do not require the protection of men and can walk out at night without a man [113].

#### 1.3.3. Employment Function Sexism (EFS)

There are still significant problems between women and men regarding employment, career orientation, and development in many countries. Even in many developed European countries, the proportion of women working in STEM (Science, Technology, Engineering, Mathematics) fields is very low [114,115,116]. There are still obstacles to the advancement of women in these areas. The concept of the “Glass Ceiling” is defined as an invisible and difficult overcome obstacle that prevents women or individuals from other races from reaching the highest level in organizations, regardless of their achievements and abilities [117]. This obstacle includes elements such as not allowing women to reach senior positions [102,118,119]. However, a small number of women who have succeeded in reaching senior positions are in human resources and research positions [120].

The restructuring of the family comes into focus as women begin to play a more significant role in employment. However, housework is still seen to be among the duties of women contributing to the home economy. Thus, sharing women’s time between paid and unpaid jobs makes it difficult to establish a work-life balance [121,122,123]. Also, gender stereotypes exist in every society. For example, while professions such as social work, teaching, dietitian, and nursing are seen as proper for women, lawyers, police, banker, engineer, and truck driver are seen as male occupations [124,125,126].

### 1.4. The Associations between Societal Gender Roles and the Occupational Choice

Society determines the perceptions about gender roles and behaviors suitable for people in many countries, including Turkey. Gender roles are effective in the professions females and males will choose or acquire in the community [32,99,105]. Some professions are defined for females, such as health and education, where women’s motherhood roles will not interfere with their home and family responsibilities. However, some professions are defined for males, such as policing, military service, and engineering, which require physical strength, courage, and intelligence [75,127,128,129,130].

The shaping of social values that affect individuals’ lives in their adulthood begins in childhood. The social environment and family are essential in forming professional choices and gender perceptions about professions that begin in early childhood [75,131,132]. Children start to research the professions of the individuals around them. Individuals’ interests and abilities, professional values, attitudes of parents and teachers, and social values attributed to professions are among the factors of this process, and gender affects this process [73,133]. Roles are distributed to females and males according to gender in the distribution of roles within the family, community, and schools. Children are expected to behave by these roles and their gender and participate in society to realize their vocational training accordingly. Unlike males, females have to devote their time out of work to activities other than production activities such as housework and family responsibilities, causing women to concentrate on specific professions or to participate less in working life [129,134].

**Figure 1:**
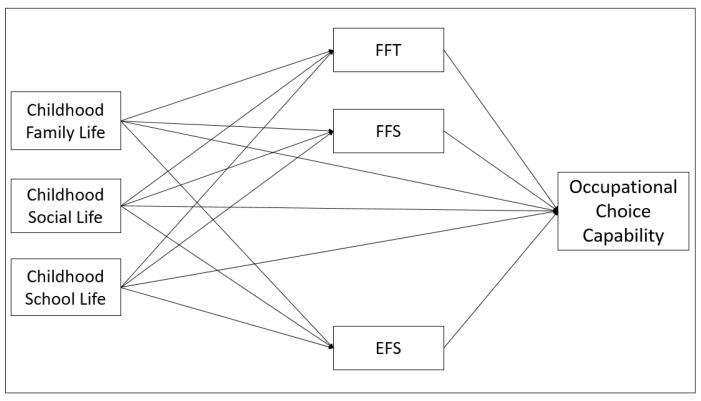
Conceptual Diagram of Model.

As depicted in Figure 1, it was assumed that family function transcendent, family function sexism, and employment function sexism had mediation roles in the association between childhood school life, one of the sub-dimensions of childhood experiences scale, and occupational choice capability.

### 1.5. Study Objectives and Hypotheses

At the beginning of the study, we asked, “How are childhood experiences and gender roles related to occupational choice capability?” To find an answer to the research question is the aim of the study. In line with the findings, implications will be made for the academy, family, and policymakers based on the associations between family, school and social life and gender roles, and career choice. For this purpose, the following hypotheses were formed based on our literature.

**Hypothesis** **1** **(H1).** *Childhood family experiences (i), social experiences (ii), and school experiences (iii) have positive relations with gender roles.*

**Hypothesis** **2** **(H2).** *Childhood experiences have a negative relation to OCCS.*

**Hypothesis** **3** **(H3).** *FFT has a mediation role in the correlation between childhood school life and OCCS.*

**Hypothesis** **4** **(H4).** *FFS has a mediation role in the correlation between childhood school life and OCCS.*

**Hypothesis** **5** **(H5).** *EFS has a mediation role in the correlation between childhood school life and OCCS.*

## 2. Method

### 2.1. Study Design, Procedure, and Participants

We used a self-report quantitative and correlational design and the questionnaire method to collect data and information in the research. The entire questionnaire, created using Google Forms, has been sent out online to school class groups and social media groups. The questionnaire form consists of 44 questions for everyone over the age of 18.

The sample for the research is people who are 18 years and over living in Turkey. A total of 805 individuals participated in the study. 65.1% of the research group was female, and 34.9% was male. The average age of the participants was 33.84 (SD = 10.7188). The education level of the individuals participating in the study is 5 (0.6%) primary school, 8 (1.0%) middle school, 45 (5.6%) high school, 446 (55.4%) university, and 301 (37.4%) of them postgraduate. The participants’ educational level was predominantly university students. The average of the income answers is 5136.93 TL (SD = 4406.16). Participants answered the question of employment status 456 (56.6%) yes, 196 (24.3%) no and 153 (19.0%) students; when asked about family attitude 187 (23.2%) were authoritarian, 266 (33.0%) were tolerant, 126 (15.7%) were unstable, 125 (15.5%) were democratic and 101 (12.5%) responded as overprotective.

### 2.2. Data Analysis

The data was converted to MS Excel for data screening and cleaning after downloading from the Google Forms website. IBM SPSS 25 and PROCESS-Macro plug-in were used for the analyses. A descriptive analysis was used to examine the demographic variables of the participants. Factor analysis was applied to the gender roles attitude scale and childhood experiences scale. Confirmatory factor analysis (CFA) was used to assess the construct validity of the components and the suitability of the measurement model depicted in Figure 1. Following the modifications, according to Kline (2016), the CFA results were found to be highly compatible with the research conceptual model, (CMIN/DF= 2.598; RMSEA = 0.045; GFI= 0.912; CFI= 0.940; TLI = 0.932; IFI= 0.940; NFI = 0.907) [135]. Demographic information of the participants was conducted by frequency analysis. For the reliability of the factors, McDonald’s omega plug-in for SPSS produced by Hayes was used [136]. Correlation analysis was performed using the Pearson coefficient to determine the relationship between variables. Multiple linear regression analysis was conducted to determine the direct effects on mediators (Model 1–3), and OCCS (Model 4–6). In the linear regression analysis, the VIF cut-off values of the independent variables should be below 3, and the tolerance cut-off values should be above 0.40. In accordance with these cut-off values, all of the VIF values were below 3, and the tolerance values were above 0.40. We used IBM SPSS 25 [137] program for correlation and direct regressions and PROCESS-Macro [138] for indirect tests. The significance level was set at 0.05.

### 2.3. Measures

The study used the Personal Information Form, Childhood Experiences Questionnaire, Gender Roles Attitude Scale, and Occupational Choice Capability Scale. All questions were entered into Google Forms and shared with individuals aged 18 and over on the internet.

#### 2.3.1. Personal Information Form

There are questions of age, gender, income level, education level, employment status, and parents’ attitudes. The answers to the question of employment status were reassessed as a dichotomous variable, with the working (coded 1) being one group and the non-working (coded 0) the other one.

#### 2.3.2. Childhood Experiences Scale

This scale was developed by Manap (2015), and the validity and reliability analysis of the scale was performed (α = 0.822) [139]. The scale consists of 12 questions and three factors, each sub-factor consists of 4 items. Questions, such as “When I was a child, my parents took care of me”, “When I was a child, I loved going to school”, and “When I was a child, I had enough friends”, were asked. Items 1–4 childhood family life, 5–8 childhood school life, and 9–12 measure childhood social life. The 5th and 7th questions are reversely coded in the scale. The scale consists of a 5-point Likert rating system [140]. An increasing score for each sub-scale means higher positive childhood experiences. McDonald’s Omega values of factors were 0.939, 0.745, and 0.920, respectively.

#### 2.3.3. Gender Roles Attitudes Scale

The scale, adapted into Turkish by Bakioğlu & Türküm (2019), was developed by [141]. The Turkish scale consists of 15 items and has a single factor. However, the Turkish version of the scale we used did not produce a single factor. Therefore, CFA was performed by using the factors from the original scale, and it was found to be compatible with three factors. FFT, FFS, and EFS factors were used in the study. Reverse questions were recoded before analysis. Questions, such as “Household chores should not be allocated by sex”, “The husband is responsible for the family so the wife must obey him”, and “Some jobs are not appropriate for women” were asked. The scale has a 5-point Likert rating (1 = I do not agree at all, 5 = I totally agree). The higher the score on the scale means the increase in the egalitarian attitude. McDonald’s omega values were 0.679, 0.678, and 0.772, respectively.

#### 2.3.4. Occupational Choice Capability Scale

This scale was developed by Vurucu (2010). The scale consists of 11 items and a single factor. The scale consists of a 5-point Likert rating system. There is no reverse-coded question. Questions such as “I think my occupation suits my abilities” and “I chose my occupation knowing the advantages and disadvantages” were asked on the scale. As the total score obtained from the scale increases, individuals’ capability in occupation choice also increases. The scoring system of the scale is 1 = strongly disagree, 2 = mostly agree, 3 = somewhat agree, 4 = agree and 5 = strongly agree. McDonald’s omega value on the scale was 0.870.

## 3. Results

### 3.1. Correlation Analysis

Among the correlation analysis made with age; low-level negative correlation with family function transcendent (r = −0.15, *p* < 0.01), low-level positive correlation with childhood school life (r= 0.09, *p* < 0.05), low-level positive correlation with childhood social life (r = 0.09, *p* < 0.05), and low-level negative correlation with OCCS (r = −0.19, *p* < 0.01) were observed, as seen in Table 1. As age increases, childhood school life and childhood social life increase, FFT, and OCCS decrease. There were associations with FFT; low-level negative correlation with FFS (r = −0.38, *p* < 0.01), low-level negative correlation with EFS (r = −0.32, *p* < 0.01) and low-level positive correlation with childhood school life (r = 0.08, *p* < 0.05). Correlation analysis with FFS; high-level positive correlation with EFS (r = 0.60, *p* < 0.01), low-level negative correlation with childhood school life (r = −0.18, *p* < 0.01), and low-level negative correlation with OCCS (r = −0.08, *p* < 0.05). There was a low-level negative correlation between EFS and childhood school life variables (r = −0.10 *p* < 0.01) association examined. There was a moderate-level positive correlation with childhood social life (r = 0.41, *p* < 0.01) and low-level positive correlation with OCCS (r = 0.18, *p* < 0.01). Low-level positive correlation with childhood social life (r = 0.17, *p* < 0.01) and low-level positive correlation with OCCS (r = 0.10, *p* < 0.01) was investigated in the correlation analysis conducted with childhood school life. A low-level positive correlation was found between childhood social life and OCCS variables (r = 0.09, *p* < 0.05).

### 3.2. Direct Regression Analysis

Table 2 shows the associations of the independent variables gender, age, education level, income, working status, childhood family life, childhood social life, and childhood school life with the mediator variables FFS (Model 1), FFT (Model 2), and EFS (Model 3). We found statistically meaningful associations between gender (B = 1.833, *p* < 0.01), childhood school life (B = −0.324, *p* < 0.01) and family function transcendent in Model 1. Age, education, income, employment status, childhood family life, and childhood social life have no significant relationships with FFT. In Model 2, the variables’ connections of gender (B = −0.843, *p* < 0.001), age (B = 0.006, *p* < 0.05), and income (B = 0.000, *p* < 0.05) were all significant with FFT. However, there were no significant associations between FFT and education, working status, childhood family life, childhood school life, and childhood social life. In Model 3, gender was significantly associated with EFS (B = 2.053, *p* < 0.001), whereas age, education, income, working status, childhood family life, childhood school life, and childhood social life weren’t significant.

Table 3, Model 4, Model 5, and Model 6 show the relationships between independents, mediators and the dependent variable. We analysed the associations of the independent variables gender, age, education level, income, working status, childhood family life, childhood social life, childhood school life, FFT, FFS, and EFS with the dependent variable OCCS. In Model 4, age (B = −0.222, *p* < 0.001), income (B = 0.00, *p* < 0.001), working status (B = 1.993, *p* < 0.01), and childhood school life (B = 1.211, *p* < 0.001) were significantly related with OCCS. Model 5 revealed that age (B = −0.221, *p* < 0.001), income (B = 0.00, *p* < 0.01), working status (B = 1.816, *p* < 0.05), and FFS (B = −0.434, *p* < 0.01) have a significant associations with OCCS. Age (B = −0,226, *p* < 0.001), income (B = 0.00, *p* < 0.05), working status (B = 1.941, *p* < 0.01), childhood family life (B = 1.527, *p* < 0.001), childhood school life (B = 1.118, *p* < 0.01), and FFS (B = −0.378, *p* < 0.05) were all statistically relevant with OCCS in Model 6.

### 3.3. Indirect Regression Analysis

We found that the childhood school life variable, an independent variable in our model, was significantly associated with the dependent variable FFS, as seen in Table 2 and Model 1. In Table 3, Model 4, the childhood school life variable was also discovered to be associated with OCCS significantly. We added the societal gender roles measure’s factors (FFT, FFS, and EFS) into previous variables after excluding the childhood experiences measure’s factors (childhood school life, childhood family life, and childhood social life) in Table 3 and Model 5.

The FFS variable was statistically significant with OCCS. In Model 6, the previous variables were combined with childhood school life and evaluated as predictors of OCCS. Childhood family life, childhood school life, and FFS variables all had statistically significant relationships with OCCS (*p* < 0.001, *p* < 0.01, and *p* < 0.05, respectively) as seen in Model 6.

The childhood school life variable continued its relationship with OCCS dependent variable through the mediator variable FFS, as seen in Table 4. So, the FFS variable had a positive and significant association as a mediator in the relationship between the childhood school life variable and the OCCS (γ = 0.1228, SE = 0.0676, 95% CI [0.0153, 0.2793]).

**Figure 2:**
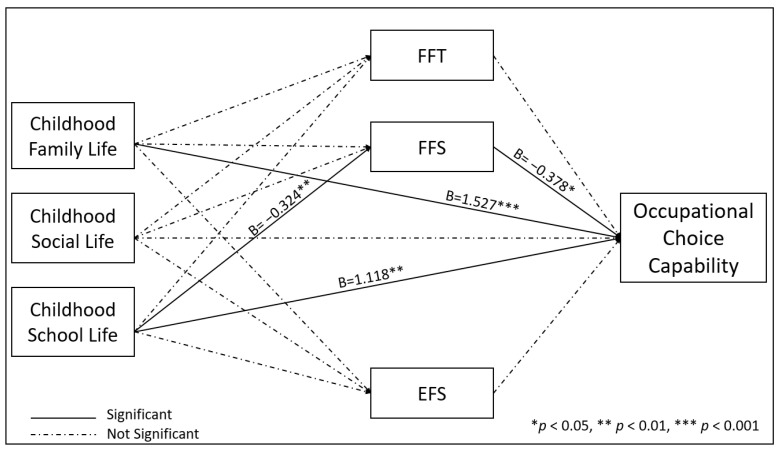
Results of the Proposed Conceptual Model.

In Figure 2, direct lines represent significant relationships, while dashed lines show no significant associations. Unstandardized coefficient values and significance levels are shown on the significant lines. In addition, the results of the hypotheses are listed in Table 5.

## 4. Discussion

The community and individuals determine gender expectations and roles, and people are expected to behave according to these determined perceptions and norms. Childhood, which is the most important time in human development processes, is when individuals’ moral values and gender perceptions begin to form. During this period, children begin to adopt the values and judgments of their families and society. These expectations that they adopt begin to manifest themselves in their adult life and affect them throughout their lives. These norms can be reflected in the profession they will choose. Career choices based on childhood experiences and society’s gender expectations can deprive employees of demonstrating their skills and abilities or allow for better performance [142]. We investigated how childhood experiences and gender roles were associated with professional choice competence. We also used age, gender, education, income, and employment status as control variables. Hypotheses H1 and H2 were partly supported, H4 was fully supported, and H3 and H5 were rejected in this study. In the H1 hypothesis, it was partially accepted as childhood school experiences (iii) had positive relations with gender roles.

Hypothesis H3 was rejected because family function transcendent (FFT) was found to have no significant association with the occupational choice capability scale (OCCS). It was expected that school life would increase the egalitarian attitude in employment function sexism (EFS), and thus OCCS would be positively related, but it did not occur, and H5 was rejected. The reason for this is that even though egalitarian attitudes increase in working life, it is seen that individuals still tend to have traditional attitudes in choosing a profession [85,102,108,126,143,144].

We examined whether or not gender roles mediated the relationship of individuals’ school life with their career decision. According to the findings, individuals’ family and school life positively and significantly related to their career choices. Family function sexism harmed their choice of profession and had a mediating role in the relationship of school life with their choice of profession. We found that males had less family function transcendent but more family function sexism and employment function sexism approaches. There were similar findings in the literature [145,146,147]. Females were egalitarian in family roles and had positive childhood school experiences, which were consistent with the literature [148,149,150,151,152]. It was thought that females take a more egalitarian attitude because they were more exposed to gender roles, had workload, and faced more discrimination [32,107,129,143]. However, some studies show that women also have a traditionalist attitude, and they see household chores as their responsibilities [89,105,115,143,150]. In our study, a positive correlation was found between age and perceived childhood school and social life, whereas a negative correlation was found with family function transcendent and occupational choice capability. This can be interpreted as the higher perception of traditionalism in family roles and lower opportunities for career choice among older individuals [146,148,153]. However, in the study of Fan & Marini (2000), it was seen that age had a positive association with egalitarian attitudes [154]. We also found that egalitarian attitude in family function transcendent had negative relationships with family function sexism, employment function sexism, and a positive relationship with childhood school life of individuals. It was seen that individuals who do not adopt an egalitarian attitude in family roles have a more traditional gender perception [129,142,155,156]. As gender roles increase in the family, traditional gender attitudes increase in employment, whereas as traditionality decreases, positive childhood social life perception and occupational choice capability increase [129]. When individuals are faced with a more egalitarian attitude within the family, their positive perceptions in their social life increase, and they can be freer in their choice of profession [149,157,158,159,160]. It was thought that individuals who grow up in a free environment, with the family’s support and a sense of responsibility, make the choices they want and are more dominant in their career choices [88,161]. While the positive perception of childhood family life increases, individuals’ perceptions of childhood social life and occupational choice capability also improves [1,162]. When childhood school life increases, people’s positiveness in childhood social life and proficiency in choosing a profession increase [163,164,165]. As individuals’ perception of positive childhood social life increases, occupational choice capability also increases. Individuals who receive sufficient support and love around them can be more competent in choosing an occupation [166,167,168].

We observed a positive correlation between occupational choice capability and working status; that is, if occupational choice capability during childhood increases, then employability during youth and adulthood tends to be better. Education level is another variable that was associated with the employability of people. Also, we found that childhood school life positively and significantly related to occupational choice capability through family function sexism. Gender roles can be consciously or unconsciously imposed on individuals in childhood by their families in a way that will direct their future professional life [155,169]. As a result of these occupational impositions, individuals either did not realize their talents and interests or did notice it too late. Attitudes such as the different professions for women and men, that women should earn less than men, that professions such as teaching and nursing are more suitable for women, and those such as engineering and piloting are more suitable for men form traditional gender perceptions [129,130,134,150]. However, we observed that individuals with positive childhood school life were less in family gender roles or those who have high family gender roles had less positive school life. Also, children who had positive school life had high competence in choosing a profession. Therefore, it is consistent with the current literature that both positive family and school life positively correlated with individuals’ future careers and professions [170,171,172,173].

At the beginning of the study, we asked a research question: How are childhood experiences and gender roles related to occupational choice capability? What distinguishes this study from other studies is how adults’ experiences in the family and school periods during childhood are associated with their current profession and career choices when they look back. It was determined that family and school experiences, especially in childhood, were correlated with the career choice competencies of individuals, and family function sexism has a significant positive mediation role in this relationship. Other remarkable findings were that family function sexism (FFS) had a significant negative association with the occupational choice capability scale (OCCS), and FFS and employment function sexism (EFS) was higher in males and family function transcendent (FFT) in females.

## 5. Study Limitations and Strengths

Research data were obtained by communicating with the groups via the internet. Therefore, a general comment cannot be made because individuals who are not in groups that do not use the internet cannot be reached. Since we used a self-report online questionnaire in the study, we could not conduct face-to-face interviews. We did not include the employment status and professions of the individuals’ parents in our study either. In future studies, we think it is important to include parents’ professions in the choice of profession. The lack of sufficient scales for studies on societal gender in Turkey is a significant problem. For this reason, not enough work has been done on the societal gender and career choice relationship in Turkey. Therefore, investigating whether gender roles affect individuals’ childhood lives and career choices differentiates our study. This study was conducted using only linear regression analysis. However, our future studies will likely produce new findings using exponential, logarithmic regression, and structural equation modeling analyses.

## 6. Conclusions and Some Implications

This study shows that individuals are affected by many factors in their choice of profession. Individuals’ proficiency levels in choosing a profession are associated with societal gender roles and childhood life experiences in family, school, and social life. We found that individuals’ positive childhood experiences and egalitarian gender attitudes help them take more conscious steps when choosing a profession that will affect their entire lives.

During childhood, school and family life have an important place in the lives of individuals. In the post-childhood career period, experiences gained in childhood affect the career process. The fact that these processes are positive, contributes to individuals being more effective and conscious about their future decisions. Children who are supported by their families, have equal opportunities regardless of gender, and are interested in school are more likely to set and strive to achieve their goals in the future. For this reason, the choice of profession, which will affect at least 40–50 years of an individual’s life, begins with the support received at school and in the family during childhood.

However, it is not enough to get support from school and family in choosing a suitable career. It is crucial for the future of both individuals and society to ensure that individuals who are free and competent in their career and profession choice, where gender roles are not imposed, add value to the society they live in. Therefore, policymakers should develop strategies considering the future effects of school and family life. These policies should create awareness about career processes and professions, in childhood, eliminate sexist approaches starting from within the family, and encourage the choice of a suitable profession for the individual.

## Figures and Tables

**Table 1:** Means, Standard Deviations and Correlations.

		Mn.	St. D.	1	2	3	4	5	6	7
1	Age	33.8	10.7	1						
2	FFT	8.29	1.89	−0.15 **	1					
3	FFS	5.68	2.72	0.02	−0.38 **	1				
4	EFS	8.09	3.44	0.01	−0.32 **	0.60 **	1			
5	C. Family life	4.01	0.97	−0.06	−0.01	0.01	0.02	1		
6	C. School life	3.83	0.94	0.09 *	0.08 *	−0.18 **	−0.10 **	0.02	1	
7	C. Social life	4.12	0.91	0.09 *	−0.04	-0.02	0.01	0.41 **	0.17 **	1
8	OCCS	36.3	10.1	−0.19 **	0.01	−0.08 *	−0.01	0.18 **	0.10 **	0.09 *

** *p* < 0.01, * *p* < 0.05, FFT = Family Function Transcendent, FFS = Family Function Sexism, EFS = Employment Function Sexism, C. = Childhood, OCCS = Occupational Choice Capability Scale.

**Table 2:** Main Regression Effects on Gender Role Attitudes Scale’s Factors.

Variable	Model 1: FFS			Model 2: FFT			Model 3: EFS		
	B	SE	*p*	B	SE	*p*	B	SE	*p*
(Constant)	5727	0.835	0.000	9.696	0.595	0.000	6.602	1.081	0.000
Gender (1–2)	1.833	0.199	0.000	−0.843	0.142	0.000	2.053	0.257	0.000
Age	−0.009	0.009	0.302	−0.015	0.006	0.021	−0.019	0.012	0.102
Education	−0.207	0.140	0.138	0.067	0.099	0.502	−0.052	0.181	0.773
Income	0.000	0.000	0.957	0.000	0.000	0.007	0.000	0.000	0.330
Working (0–1)	−0.010	0.188	0.957	−0.210	0.134	0.118	0.297	0.244	0.224
C. Family Life	0.034	0.103	0,741	0.021	0.073	0.770	0.000	0.134	0.998
C. Social Life	−0.049	0.111	0.655	−0.068	0.079	0.386	0.058	0.143	0.687
C. School Life	−0.324	0.099	0.001	0.121	0.071	0.087	−0.180	0.129	0.163
F		15.14			10.24			9.90	
p		<0.001			<0.001			<0.001	
R2		0.132			0.093			0.090	

C. = Childhood, For Working 0 = No 1 = Yes, For Gender 1 = Female 2 = Male, FFS = Family function sexism, FFT = Family function transcendent, EFS = Employment function sexism.

**Table 3:** Main Effects on Occupational Choice Capability Scale (OCCS).

Variable	Model 4: OCCS			Model 5: OCCS			Model 6: OCCS		
	B	SE	*p*	B	SE	*p*	B	SE	*p*
(Constant)	27.260	3144	0.000	39,119	3601	0.000	30,268	3938	0.000
Gender f–m (1–2)	0.989	0.749	0.187	1.389	0.801	0.083	1.421	0.795	0.074
Age	−0.222	0.034	0.000	−0.221	0.034	0.000	−0.226	0.034	0.000
Education	0.284	0.526	0.589	0.812	0.524	0.122	0.218	0.526	0.679
Income	0.000	0.000	0.013	0.000	0.000	0.009	0.000	0.000	0.015
Working 0–1	1.993	0.710	0.005	1.816	0.723	0.012	1.941	0.711	0.006
C. Family Life	1.512	0.389	0.000				1.527	0.388	0.000
C. Social Life	0.269	0.418	0.520				0.237	0.417	0.569
C. School Life	1.211	0.374	0.001				1.118	0.376	0.003
FFT				−0.127	0.203	0.531	−0.136	0.199	0.496
FFS				−0.434	0.167	0.009	−0.378	0.165	0.022
EFS				0.085	0.127	0.502	0.072	0.125	0.565
F		11.20			7.79			8.70	
p		<0.001			<0.001			<0.001	
R2		0.101			0.073			0.108	

C. = Childhood, For Working 0 = No 1 = Yes, FFT = Family Function Transcendent, FFS = Family Function Sexism, EFS = Employment Function Sexism, OCCS = Occupational Choice Capability Scale.

**Table 4:** Total, Direct, and Indirect Analysis on OCCS.

					Unstd.	SE	LLCI	ULCI	
Total Effect of Childhood School Life on OCCS					1.211	0.3744	0.4760	1.946	Sig.
Direct Effect of Childhood School Life on OCCS					1.117	0.3765	0.3786	1.856	Sig.
Path		Indirect Effects			Unstd.				
CSL	>	FFT	>	OCCS	−0.0165	0.0302	−0.0863	0.0364	N.S.
CSL	>	FFS	>	OCCS	0.1228	0.0676	0.0153	0.2793	Sig.
CSL	>	EFS	>	OCCS	−0.0128	0.0291	−0.0837	0.0388	N.S.

CSL = Childhood School Life, Sig. = Significant, N.S. = Not Significant.

**Table 5:** Summary of Hypotheses Testing Results.

No.	Relationship	Proposed Relationship	Support
H1	Childhood Experiences–Gender Roles	Positive	Yes (Partly)
H2	Gender Roles–OCCS	Negative	Yes (Partly)
H3	Childhood School Life–OCCS	Mediated by FFT	No
H4	Childhood School Life–OCCS	Moderated by FFS	Yes
H5	Childhood School Life–OCCS	Moderated by EFS	No

## Data Availability

The data presented in this study are available on request from the corresponding author.
